# The Treatment of Ankylostoma Anæmia

**Published:** 1914-07-25

**Authors:** 


### The Treatment of Ankylostoma Anæmia.

The ravages of hookworm disease are fortunately almost unknown in Great Britain, though competent observers believe that only a series of lucky accidents saved us a few years ago from an outbreak. In Egypt and in the United States it is a very serious cause of ill-health; and Professor Day, of Cairo, has formulated in the *Lancet* his conclusions as to the best treatment of the anæmia which is the principal symptom of ankylostoma infection. (1) Patients with slight anæmia may benefit from hæmatinics without the expulsion of worms. But no case of decided anæmia can be really improved unless the worms are expelled. (2) Recent cases of moderate anæmia from ankylostomiasis are cured by expulsion of the worms. Recovery in most chronic cases is very slow unless hæmatinics be given. (3) Under treatment with vermicides and iron the rise in hæmoglobin is a reliable index to the expulsion of the worms, provided the marrow be in a condition to respond. (4) The administration of simple forms of iron by the mouth is more satisfactory than the use of organic compounds or of hypodermic medication. (5) Manganese cannot be substituted for iron. (6) After the expulsion of worms in a moderately severe case the recovery from anæmia is better and quicker when arsenic is given as well as iron. (7) In severe cases of ankylostomiasis the administration of arsenic is often essential to recovery. (8) In such cases arsenic given by the mouth may be ineffectual, but hypodermic medication usually succeeds. (9) Arsenic by itself is useless. Iron is always essential to recovery from severe ankylostomiasis. (10) Persistent eosinophilia after the complete expulsion of worms may be attributed to the presence of living larvæ in the tissues. (11) Intense and persistent anæmia in ankylostomiasis generally denotes exhaustion and atrophy of the bone marrow.

